# The enhancing effects of testosterone in exposure treatment for social anxiety disorder: a randomized proof-of-concept trial

**DOI:** 10.1038/s41398-021-01556-8

**Published:** 2021-08-20

**Authors:** Moniek H. M. Hutschemaekers, Rianne A. de Kleine, Gert-Jan Hendriks, Mirjam Kampman, Karin Roelofs

**Affiliations:** 1 Overwaal Centre of Expertise for Anxiety Disorders, OCD and PTSD, Pro Persona Institute for Integrated Mental Health Care, Nijmegen, The Netherlands; 2 Behavioural Science Institute, Radboud University, Nijmegen, The Netherlands; 3 Institute of Psychology, Leiden University, Leiden, The Netherlands; 4 Department of Psychiatry, Radboud University Medical Center, Nijmegen, The Netherlands; 5 Donders Institute for Brain, Cognition and Behaviour, Centre for Neuroimaging, Radboud University, Nijmegen, The Netherlands

**Keywords:** Human behaviour, Predictive markers

## Abstract

Individuals with a social anxiety disorder (SAD) show hypofunctioning of the hypothalamus–pituitary-gonadal (HPG) axis, which is linked to social fear and avoidance behavior. As testosterone administration has been shown to facilitate social-approach behavior in this population, it may enhance the effectiveness of exposure treatment. In this proof-of-concept study, we performed a randomized clinical assay in which 55 women diagnosed with SAD received two exposure therapy sessions. Session 1 was supplemented with either testosterone (0.50 mg) or placebo. Next, transfer effects of testosterone augmentation on within-session subjective fear responses and SAD symptom severity were assessed during a second, unenhanced exposure session (session 2) and at a 1-month follow-up, respectively. The participants having received testosterone showed a more reactive fear pattern, with higher peaks and steeper reductions in fear levels in session 2. Post-hoc exploration of moderating effects of endogenous testosterone levels, revealed that this pattern was specific for women with high basal testosterone, both in the augmented and in the transfer session. In contrast, the participants with low endogenous testosterone showed reduced peak fear levels throughout session 1, again with transfer to the unenhanced session. Testosterone did not significantly affect self-reported anxiety. The effects of testosterone supplementation on fear levels show transfer to non-enhanced exposure, with effects being modulated by endogenous testosterone. These first preliminary results indicate that testosterone may act on important fear mechanisms during exposure, providing the empirical groundwork for further exploration of multi-session testosterone-enhanced exposure treatment for SAD.

## Introduction

With a lifetime prevalence of 13% and long-term disability, social anxiety disorder (SAD) is the most common and burdensome of all anxiety disorders [1–3]. Persistent avoidance is the main factor hindering the extinction of fear during social situations, which is why reducing avoidance behavior is the core target of exposure therapy, the treatment of choice for SAD [4, 5]. However, with response rates of 45–55%, the intervention leaves room for improvement [6–9]. Particularly, augmentation strategies aimed at alleviating social avoidance and promoting social approach have the potential to boost core mechanisms assumed to underlie the effects of exposure in SAD.

Previous attempts to enhance the therapy’s efficacy with pharmacological agents (e.g., d-cycloserine (DCS), yohimbine, oxytocin) targeted the process of extinction learning in SAD [10–15]. Although the results are encouraging, no pharmacological enhancer has been tested that directly acts on acute within-session social-approach behavior, essential for effective exposure.

Testosterone, the end product of the hypothalamus–pituitary–gonadal (HPG) axis, is important in the regulation of social motivational behavior, including approach and avoidance behavior [16]. In both animal and human studies, low endogenous testosterone has been linked to socially submissive, anxious, and avoidant behaviors [17–19], while high basal testosterone is related to social dominance and approach behavior [20, 21]. Importantly, in individuals with SAD [22] and other social avoidance-related disorders such as depression [22, 23] reduced levels of endogenous testosterone have been found. Moreover, relatively high pre-treatment testosterone concentrations predicted a better outcome of exposure therapy in terms of larger symptom reductions [24]. Additionally, causal studies on the relationship between testosterone and avoidance behavior further confirm the social avoidance-reducing and approach-facilitating properties of testosterone [25–27]. Testosterone acts on dopaminergic projections from the amygdala to the striatum and its administration was shown to bias amygdala activity toward social threat approach in humans [28, 29]. At a behavioral level, testosterone administered to healthy participants prior to threat exposure was found to reduce fear, enhance reward sensitivity, and promote social-approach motivation [25, 26, 30]. Eye-tracking and electrophysiological studies by our group showed that, when administered to women with SAD, testosterone reduced automatic threat bias to angry faces [31, 32], diminished social avoidance, and promoted prosocial behavior, including approach toward angry faces [27]. Although preliminary, these findings provide promising evidence that testosterone administration prior to exposure therapy enhances the treatment’s efficacy by promoting social approach, one of its main goals [24, 33].

Given its profound role in the regulation of social motivational behavior, in the present proof-of-concept study, we test the augmentation effects of testosterone on exposure in women coping with SAD in a randomized clinical assay, comparing a single testosterone-enhanced session (0.50 mg) with a placebo-supplemented session. To assess the transfer of testosterone-induced effects, the participants engaged in a similar but unenhanced exposure session one week later. We hypothesized that, compared to the placebo group, testosterone-enhanced exposure would induce steeper reductions in subjective fear during the repeated exposure session, as an index of retention of extinction learning during session 1 [34–36]. In addition, we verified whether testosterone supplementation would affect self-reported pre-to-post-treatment social anxiety symptoms. Finally, we explored the physiological effects of acute testosterone administration by assessing within-session HR. Given the recent insight into the moderating effects of endogenous testosterone on exogenous testosterone [37–39] and the efficacy of exposure treatment for SAD in particular [24], we explored endogenous testosterone as a moderating factor in our analyses.

## Materials and methods

### Participants

Participants were recruited from an outpatient clinic specializing in the treatment of anxiety disorders, from the Radboud University Nijmegen, and from the community from 2017 through 2019. Inclusion criteria were: (1) woman, (2) age: 18–45 years, (3) primary diagnosis of SAD (as assessed with the Mini International Neuropsychiatric Interview (MINI; [40]), with a predominant fear of public speaking, and (4) score >30 on the Liebowitz Social Anxiety Scale (LSAS; [41]). We focused exclusively on women because the pharmacodynamics of the currently used testosterone administration methods have as yet been established in women only [42]. Exclusion criteria were: (A) prior non-response to speech exposure therapy for SAD, (B) other predominant mental disorder(s), (C) (current or lifetime) psychosis or delusion disorders, (D) significant suicidal ideation or behavior within 6 months prior to screening, (E) intellectual disability, (F) substance or alcohol dependence, (G) somatic illness, H) unwillingness to use an active form of birth control during the trial, (I) pregnancy or lactation, (J) infertility, (K) antipsychotic medication, (L) unstable regimen of antidepressants or benzodiazepines within 6 weeks prior to enrollment, (M) insufficient proficiency in the Dutch language, (N) current use of contraceptives containing cyproterone acetate. All participants received 70 Euros for their participation. Ethical approval for this study was granted by the local (Arnhem-Nijmegen) Review Board.

In total, 55 women suffering from SAD (*M*_age_ = 23.31, SD = 5.63, range = 18–43) were included in the study sample. One participant dropped out before the first exposure session (due to illness). She was replaced by another participant to ensure equal group sizes, resulting in 55 participants receiving the allocated drug (testosterone/placebo) and 54 the exposure sessions: 27 per group (see Supplementary Fig. S1 for CONSORT flowchart). After study completion, one participant (testosterone group) divulged she had been on atypical antipsychotic quetiapine, which fact she had not mentioned during eligibility screening. Since she had consistently used a low, stable dose (25 mg) for the last 18 months, we decided not to exclude her from the analyses.

### Medication and randomization

The pharmacist providing the study solutions randomly assigned participants to testosterone (T) or placebo (P) in blocks of four (no stratification). T was suspended in a clear solution (0.5 ml) with 0.5 mg hydroxypropyl-beta-cyclodextrin, 0.005 ml ethanol 96%, and distilled water. P contained the same ingredients, barring T. Participants held the liquid under their tongues for 60 seconds. In women, this dose yields a sharp increase in plasma testosterone concentrations within 15 min and declines to baseline within 90 min [43]. Pharmacodynamic effects can be assayed 4–6 h after intake [30, 42]. Researchers, therapists, and participants were blinded to the group allocation until the completion of the primary outcome analyses.

### Exposure intervention

The participants engaged in two 90-min public-speaking exposure sessions delivered one week apart in accordance with the protocols developed by Rodebaugh and colleagues [10, 13]. The sessions were standardized with respect to exposure length (6–8 min), preparation time (max. 5 min), reaction of the experimenter (neutral), and the availability of notes and speech topic. On the morning of the first day, the participants received psychoeducation about SAD and exposure, with the first session starting after 4 h. In both sessions, psychoeducation was repeated and personalized harm expectancies and goals were assessed. Then, the participants presented their prepared speech in front of a therapist, two confederates, and a camera. They reviewed their videotaped performance afterward together with the therapist. The therapists were psychology students in their last year of training (BA and MA level) trained and supervised by experienced, board-certified psychologists (M.H.M.H. and M.K). To guarantee adherence to the protocol, the therapists were instructed to fill out a checklist of all protocol components and to report any deviations from the protocol. The checklists and reports on deviations showed that 96.3% of the sessions were delivered in accordance with the protocol.

### Outcome measures

#### Within-session fear (primary outcome)

Participants rated their fear levels on a subjective units of distress (SUD) scale ranging from 0: No fear to 100: Extreme fear [44]. SUDs were collected after psychoeducation (initial SUD), immediately prior to each exposure session (baseline SUD), immediately prior to the speech (start SUD), every 2 min during, and immediately after the speech (endpoint SUD).

#### Symptom severity (secondary outcome)

Social anxiety symptoms were assessed with the Social Phobia Scale (SPS; [45]), a self-report measure assessing the fear of being observed or watched during social or performance situations. The scale has shown good internal consistency ([45]; *α* = 0.94; Dutch translation; [46], *α* = 0.91, current study *α* = 0.86). Participants completed the SPS at baseline, after the second exposure session (post-treatment) and at the 1-month follow-up (FU) assessment.

#### Saliva samples

**Fig. 1:**
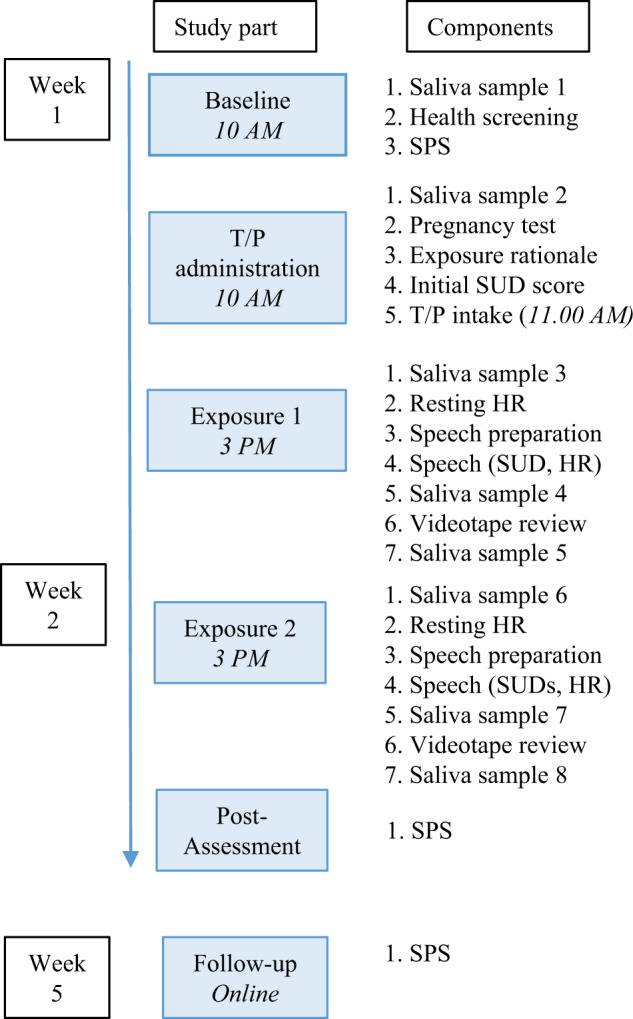
Timing and procedure of the study protocol. SPS Social Phobia Scale, SUD subjective units of distress, T testosterone, P placebo, HR heart rate. Since pregnancy was a reason for exclusion, the pregnancy test was to ascertain that none of our participants was pregnant prior to the start of the testosterone-enhanced session.

To determine endogenous testosterone levels, saliva samples were collected (2 ml passive drool saliva by Salicap; Hamburg, Germany) at eight time points (Fig. 1): (1) at baseline, (2) prior to T/P intake, (3) prior to exposure session 1, (4) immediately after speech delivery in session 1, (5) 30 min after speech delivery in session 1, (6) prior to exposure session 2, (7) immediately after speech delivery in session 2, and (8) 30 min after speech delivery in session 2. Participants were asked to conform to certain directives regarding food and drink intake to prevent pollution of the saliva samples. Samples were stored at −20 °C until radio-immune assays were performed at Dr. Kirschbaum’s laboratory (Dresden, Germany); for descriptions of the methodology, see refs. [47, 48].

#### Procedure

After having provided their informed consent, participants were screened online for eligibility, to which end they filled out the LSAS and answered general screening questions (e.g., age, treatment status, infertility, menstrual cycle). Eligible participants were telephoned for further screening (MINI, check in/exclusion criteria), after which they learned whether they would be participating in the study (see supplement for details). All other assessments and the exposure sessions took place at the treatment facility. After enrollment (see Fig. 1 for timing and procedure), participants completed the baseline assessment^1^, with the first exposure session being scheduled within one week. The morning of the session, the participants took a pregnancy test, saliva was collected, and psychoeducation provided, after which the participants completed a non-speech-related SUD and received T/P (administered by a research assistant). After 4 h, during which time participants were instructed to avoid physically and psychologically straining activities and heavy meals, they returned for the exposure session, before which saliva was collected and resting HR was recorded. Another saliva sample was collected immediately upon completion of the speech and 30 min thereafter. During the speech, SUDs and HRs were collected. At the end of the session, the therapist checked for adverse drug effects by asking participants about physical complaints. A week later, the second exposure session took place, with all steps being identical to those of the first session barring supplement administration. After a 30-min break, participants took the post-exposure assessment comprising the SPS and a computer task (reported elsewhere). We asked all participants to refrain from using alcohol, drugs, or medication (except from their stable dose of psychotropic medication) during testing days. One month later, participants once again completed the SPS online. This study was registered in the Dutch Trial Register: https://www.trialregister.nl/trial/6238 and at EudraCT (2014–004475–23).

### Statistical analyses

To test the effects of testosterone augmentation on subjective fear, we used mixed models. A sample size of 52 participants was deemed necessary to detect group differences with at least a moderate effect size and a power of 80%. We tested the acute effects of the enhancement (session 1) and transfer to unenhanced exposure (session 2) separately. Its effects on SAD symptoms (SPS scores) were tested in an additional model (see below). Moreover, we explored augmentation effects on HR in similar models as subjective fear (see supplementary materials for details). We used the Lme4 package in R [49] and *p*-values were calculated using the likelihood ratio tests (Afex package) [50]. Independent continuous predictors were centered and sum-to-zero contrasts used. Consistent with the recommendations for mixed models [51], we report unstandardized effect sizes (estimates).

More specifically, to determine whether the added testosterone had affected self-reported fear (SUDs) during session 1, group (T/P) and time (start, 2 min, 4 min, 6 min, 8 min, end) were included as predictors. Linear, quadratic, and cubic time terms were modeled since we expected that the SUD scores would not necessarily follow a linear pattern (e.g., they could increase first [fear activation] before they decrease). Participant was included as random intercept and time (linear, quadratic, cubic) as random slope. Initial SUDs (rated after psychoeducation) were included as a fixed factor to control for variance unrelated to time or group; see also ref. [13]. To examine whether enhanced exposure affected fear patterns during the second unenhanced session, we ran the same analysis used for the first session. As regards the effects of the enhancement on symptom severity, we modeled SPS scores, with group (T/P) and time (pre/post/FU) as predictors and participant as the random intercept.

Finally, in light of recent insights into the role of endogenous testosterone on the effects of exposure treatment for SAD [24], we conducted post-hoc tests, re-running all our analyses now including basal testosterone levels (mean samples 1 and 2). Since we detected some outliers (visual inspection of boxplots) in the baseline data, we repeated the analyses after winsorizing (i.e., setting extreme baseline testosterone values to the second and 98th percentile to thus reduce the effects of spurious outliers). The results remained unchanged. Also, given that age and hormonal birth control are known factors affecting endogenous testosterone, we checked if the observed effects would hold after correcting for these variables in all models.

## Results

### Attrition

One participant receiving placebo dropped out before the first exposure due to illness (see participant section). Another participant in the same group dropped out during the first session (3.6%). All other participants completed both sessions and the follow-up.

### Sample characteristics

The data of 54 participants were analyzed (*M*_age_ = 23.31, SD = 5.64, range = 18–43; Table 1). There were no significant between-group differences on any of the baseline measures. The manipulation was successful: compared to the placebo group, testosterone levels after testosterone administration (sample 3) were significantly higher in the enhanced group, moreover blinding was successful, participants were unaware if they received T or P (Table 1).

**Table 1:** Sample characteristics (*N* = 54).

	Total sample *(N* = 54) Mean (SD)	Placebo *(n* = 27), Mean (SD)	Testosterone *(n* = 27) Mean (SD)	*t* or *χ*^2^, *p*
*Demographics*				
Age	23.31 (5.64)	24.00 (6.85)	22.61 (4.12)	0.90, *0.372*
SPS total score	30.20 (11.85)	32.15 (13.82)	28.26 (9.35)	1.21, *0.232*
LSAS total score	63.06 (19.24)	64.70 (18.96)	61.41 (19.73)	0.62, *0.534*
Initial SUD score	20.59 (13.86)	20.44 (11.33)	20.74 (16.21)	0.77, *0*.*938*
Testosterone sample 1	15.17 (16.41)	15.94 (19.65)	15.47 (12.76)	0.105, *0*.*917*
Testosterone sample 2	16.21 (15.45)	17.57 (16.83)	14.80 (14.06)	0.650, *0.518*
Testosterone sample 3	252.49 (380.03)	15.29 (16.19)	489.68 (421.06)	−5.85, *<0.001*
Educational level (*n*)				1.15, *0.680*
High school	7	2	5	
Intermediate vocational	6	3	3	
Higher vocational	11	6	5	
University	30	16	14	
Psychological treatment (*n*)	9	5	4	0.13, *0.715*
Psychotropic medication use (*n*)	4	2	2	2.00, 0.572
Contraceptive use (*n*)				1.29, *0*.*733*
Hormonal	30	15	15	
Non-hormonal	24	12	12	0.00*, 1.00*
Comorbid anxiety disorder (*n*)	18	11	7	1.39, *0*.*239*
Comorbid depressive disorder (*n*)	5	2	3	0.22, *0.639*
Participants believed to be in T group (*n*)	12	7	5	

For testosterone sample 2 in the testosterone group, *n* = 26 as one participant had a missing value. Testosterone values are in picograms per milliliter (pg/ml). (*n*) = values are expressed as number of participants.

*SPS* Social Phobia Scale, *LSAS* Liebowitz Social Anxiety Scale, *SUD* subjective units of distress.

### Adverse events

The testosterone and placebo arms did not differ with respect to adverse events; no serious events were reported in either group (for details see supplement).

### Acute effects of testosterone augmentation (session 1)

#### Fear

Before reporting on the critical transfer session (2), we first describe the acute effects of testosterone on fear scores in session 1. Fear scores decreased over time (linear and quadratic), with exposure resulting in the expected within-session reduction: Estimate(linear) = −81.96 (16.76), *F*(1,51) = 23.89, *p* < 0.001, Estimate(quadratic) = −85.12(13.99), *F*(1,51) = 36.95, *p* < 0.001. The interaction between time (linear or quadratic) and group was not significant: *p*-values > 0.618 (for details see supplement and Supplementary Fig. S2A).

**Fig. 2:**
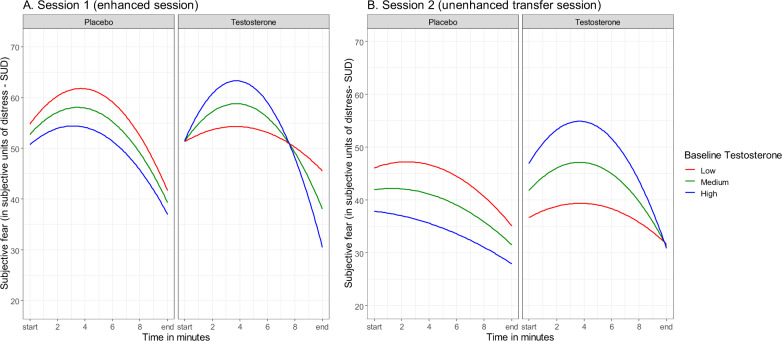
Subjective fear levels during exposure as a function of baseline testosterone per group (T/P). The figure illustrates the evolution of subjective fear levels during exposure with placebo (P) or testosterone (T) (session 1; **A**) and unenhanced exposure indicating transfer after P and T (session 2; **B**). Fear, expressed in subjective units of distress (SUDs), is displayed over time as a function of baseline-T. In order to visualize the interaction effect between baseline-T and time, we subdivided baseline-T into low (−1 SD), medium (mean), and high (+1 SD) values. Thus, the plot shows model-based predicted values, illustrating that high baseline-T is associated with higher SUD reactivity during testosterone-enhanced exposure (session 1), a pattern that largely transfers to the second unenhanced exposure (session 2). Note that for both groups there is no correlation between baseline-T and start SUDs in session 1 (*r*_placebo_ = −0.07, *p* = 0.73; *r*_testosterone_ = 0.06, *p* *=* 0.788) or session 2 (*r*_placebo_ = −0.03, *p* = 0.873; *r*_testosterone_ = 0.14, *p* *=* 0.472), indicating that effects are not driven by differences in start SUDs as a function of baseline-T but merely reflect differences in within-session fear patterns.

In the post-hoc model including baseline testosterone levels, the effects of time were confirmed: *p*-values < 0.049. There was no significant time × group interaction, *p-*values > 0.562 (see Supplementary section), but the effect for time(quadratic) × group × baseline-T effect was significant: Estimate = 2.26(0.94), *F*(1,48) = 5.72, *p* *=* 0.021. As to fear patterns as a function of endogenous testosterone, in the placebo group, fear was not moderated by baseline testosterone: Estimate = 0.82(1.14), *F*(1,24) = 0.52, *p* *=* 0.476. In contrast, in the testosterone group, fear patterns in the participants with higher baseline testosterone were relatively more reactive, showing higher peaks followed by stronger reductions, than in those with lower values, where fear responses were characterized by relatively blunted peaks followed by weaker reductions: Estimate = −3.73(1.48), *F*(1,24) = 6.32, *p* *=* 0.019 (Fig. 2A). Inclusion of age or hormonal contraceptives did not improve the fit of any of the models, so these were dropped from the analyses.

### Transfer effects of testosterone augmentation (session 2)

#### Fear

Next, we tested effects for the critical unenhanced session (2). Fear reduced over time^2^: Estimate(linear) = −62.95(15.42), *F*(1,50) = 16.66, *p* < 0.001, Estimate(quadratic) = −48.32(11.03), *F*(1,50) = 19.18, *p* < 0.001, Estimate(cubic) = −36.76(8.90), *F*(1,50) = 17.01, *p* < 0.001. Critically, there was a group × time(quadratic) interaction: Estimate = 23.68(11.03), *F*(1,50) = 4.61, *p* *=* 0.037. Compared to participants having received placebo, the participants in the testosterone group showed a more reactive fear pattern (higher SUDs) with a steeper decline at the end of the session (Supplementary Fig. S2B).

The post-hoc observation that testosterone administration had resulted in steeper fear reductions in participants with high baseline testosterone (session 1) was again made in the second, non-enhanced session, with fear levels showing a similar time(quadratic) × group × Baseline-T interaction: Estimate = 1.53(0.74), *F*(1,47) = 4.22*, p* *=* 0.045. In the placebo group, session-2 fear levels followed the same quadratic pattern regardless of baseline testosterone: Estimate = 0.77(0.82), *F*(1,23) = 0.94, *p* *=* 0.357. In the testosterone group, they showed higher peaks followed by stronger reductions for participants with high baseline testosterone, whereas for those with low baseline testosterone peak fear levels flattened: Estimate = −2.29(1.27), *F*(1,23) = 3.26, *p* *=* 0.084 (Fig. 2B).

#### Post-hoc exploration of heart rate in sessions 1 and 2^3^

Our results so far suggest that testosterone may have an acute impact on exposure mechanisms, boosting a steeper fear-decline in individuals with high baseline testosterone levels in session 1, which could be relevant for the subsequent transfer to session 2. To deepen our understanding of potential mechanisms affected during session 1, we post-hoc explored whether psychophysiological reactivity (HR) mimics the acute effects of testosterone administration on fear levels. HR patterns largely mimicked those of the subjective fear patterns in session 1: There was a non-significant trend toward a time(linear) × group × baseline-T interaction: Estimate = 0.80(0.42), *F*(1,44) = 3.64*, p* *=* 0.063. In the placebo group HR decline followed the same slope regardless of baseline testosterone: Estimate = 0.04(0.03), *F*(1,23) = 0.056, *p* = 0.461, while in the testosterone group HR reduced more for the participants with higher baseline testosterone levels: Estimate = −0.12(.06), *F*(1,21) = 3.89, *p* = 0.061. These acute psychophysiological effects did not transfer to the non-enhanced transfer session, indicating that they may support the acute fear reactivity, but that it is the subjective fear pattern that is longer-term affected (for full analyses see supplement and Supplementary Fig. S3).

### The effect of testosterone administration on social anxiety symptoms

SAD symptoms decreased from pre- to post-treatment (*M*_pre_ = 30.20 vs. *M*_post_ = 28.04), Estimate = −2.22(1.23), *t*(1,102) = −1.80, *p* *=* 0.074 and significantly so from pre- to FU: Estimate = −7.02(1.23), *t*(1,102) = −5.70, *p* < 0.001 (*M*_pre_ = 30.20, *M*_FU_ = 23.26). There was no group × time interaction: pre-post, Estimate = −0.48(1.23), *t*(1,102) = −0.39, *p* = 0.697, pre-FU: Estimate = −2.10(1.23), *t*(1,102) = −1.70, *p* *=* 0.092, indicating that symptom severity after treatment discontinuation did not show any of the effects of testosterone enhancement on within-session fear. There was no effect of baseline testosterone.

## Discussion

Seeking to test the effects of testosterone-augmented exposure treatment for individuals with SAD, we compared a single exposure session with testosterone supplementation (0.50 mg) to an exposure session with placebo, assessing fear levels in an unenhanced second session and SAD severity after one month. The exposure sessions were successful in reducing fear, HR, and SAD symptoms, independent of group. Foremost, testosterone augmentation was associated with higher peaks followed by a steeper decline in fear at the end of the second unenhanced session. Post-hoc analyses revealed this pattern was most pronounced in participants with higher baseline testosterone and evident in both the enhanced (session 1) and the transfer session (session 2). Peak fear levels in the participants with low basal testosterone remained lower throughout both sessions. Testosterone enhancement did not significantly change SAD symptom severity. Our proof-of-concept results provide preliminary support that testosterone may act on important mechanisms of exposure, meriting further examination of multiple-session testosterone-enhanced exposure therapy for SAD.

The effects of testosterone partly coincide with several studies supporting avoidance-reducing and social-approach-facilitating properties of the hormone [25–27]. Moreover, the SUD patterns (increase prior to a decrease) are in line with Emotional Processing Theory (EPT) positing that fear needs to be activated first, and only after prolonged exposure, fear levels will drop. Such reactive pattern is deemed essential for learning and, hence, transfer in the long run [34, 35]. By boosting initial engagement with the feared stimulus, testosterone may affect important learning mechanisms reinforcing transfer (e.g., initial engagement to the feared stimulus in session 1 that transfers to fear levels in a second unenhanced session). Such interpretation is in line with the threat-approach boosting effects of testosterone in patients with social anxiety disorder [27, 31].

Then again, acute testosterone-augmentation effects depended on endogenous testosterone levels. This is consistent with evidence showing that individual differences in basal testosterone and proxies of fetal testosterone exposure (2D:4D ratio) moderate the effects of exogenous testosterone on various pertinent behavioral processes, including social approach, aggression, dominance, and risk-taking [37–39, 52, 53].

The interaction between endogenous testosterone levels and exogenous testosterone administration was interesting and deepen our understanding of the primary results, in that exclusively the participants with low baseline concentrations having received testosterone reported blunted peak fear levels. This is in line with earlier findings regarding the anxiolytic properties of testosterone [30, 54]. Together, these findings suggest that women with relatively low endogenous testosterone show lowered threat response following testosterone supplementation that transfers to the non-enhanced session. In contrast, although the women with higher basal testosterone reported similar fear levels at the end of the enhanced session, they arrived there via a different, more fear-reactive route that appeared to be transferred to the unenhanced session. Arguably, the testosterone-induced effects (e.g., higher peak fear) in women with higher endogenous levels could be interpreted as negative. However, in theoretical accounts of exposure therapy (i.e., EPT [35] and inhibitory learning theory [36, 55]) high fear levels during (initial) exposure sessions are deemed beneficial for a good response, prompting the hypothesis that, it may facilitate essential exposure mechanisms in those with high basal levels. In the present proof of concept study, we cannot yet verify such qualification of patterns as beneficial or not, particularly as our single session-enhancement did not result in lower SUD levels at the end of the second exposure, in the testosterone compared to placebo group. We can only make speculations based on theoretical grounds and clearly, treatment protocols with more exposure sessions are needed to further elucidate the effects of exogenous testosterone on fear activation and reduction within and across exposure sessions.

That endogenous testosterone moderates the effects of exogenous testosterone may be explained by trait factors, including individual differences in the sensitivity of the androgen receptor (AR), where relative AR insensitivity has been reported for people with low basal concentrations [56, 57]. Moreover, testosterone administration can lead to AR downregulation in hypogonadal mice and human males, while long-lasting effects of endogenous testosterone may upregulate its expression [58].

The observed effects of exogenous testosterone on fear levels did not generalize to SAD symptoms. Although we extend previous observations that a single dose of testosterone can affect threat-approach behavior in SAD in an experimental context [27, 31], to fear-reactivity in a clinical context, we do not observe an effect on clinical outcomes. This may be a result of the fact that our symptom outcome measure (SPS) only has one item measuring speech anxiety. We recommend future studies to use a measure more sensitive to changes in speech anxiety. On the other hand, research testing other pharmacological enhancers demonstrated that repeated doses yielded better exposure outcomes than did a single dose [14, 59] So, future investigations comprising more testosterone-enhanced sessions are necessary to establish whether testosterone can improve SAD symptoms.

As to the strengths of our study, we can say that with a comparative randomized clinical assay we were able to establish that the administration of a single dose of testosterone was safe and tolerable; there were no adverse events or augmentation-related drop-out. Moreover, by comparing effects in two successive sessions, we were able to examine the direct effects of the enhancement and their transfer in a relatively quick and cost-effective manner. However, since we only included women because the administration method we used has as yet only been applied in women [42], we cannot say whether our findings will generalize to men. Also, due to inclusion restrictions (e.g., birth control types, pregnancy) and because women with relatively low endogenous testosterone were relatively underrepresented, it remains to be tested whether findings generalize to a broader group and replication in a larger, more varied sample is needed. Furthermore, although all our participants met the SAD criteria, their baseline severity scores were somewhat lower than those reported in other exposure enhancement studies [11, 14, 60]. Even though our findings show that exogenous testosterone already exerts effects in a population with relatively mild symptoms, it needs to be shown whether they generalize to more severely impaired populations.

To conclude, testosterone-augmented exposure differentially affects in-session fear levels, partly depending on baseline testosterone levels of individuals with SAD. It reduced self-reported peak fear levels in individuals with low baseline testosterone, and increased reactive patterns in individuals with high baseline testosterone. Because both patterns may be relevant for long-term extinction learning, we hope this study inspires an investigation of the longer-term effects of repeated testosterone-enhancements in SAD.

## Supplementary information

Supplementary materials

Figure S1

Figure S2

Figure S3

## Footnotes

1 Computerized tasks were also part of the baseline assessment, but outcomes will be reported elsewhere.

2 The residuals of the models for the SUDs in session 2 and the SAD symptom scores showed one standardized value >3; therefore, the models were re-run without this outlier. Since our primary outcomes were similar, the results presented include all data points.

3 We initially modeled linear, quadratic, and cubic time terms in all HR analyses but dropped the cubic term as it did not improve the model fit.
