# Supplementary material for: The enhancing effects of testosterone in exposure treatment for social anxiety disorder: a randomized proof-of-concept trial
Source: Transl Psychiatry. 2021 Aug 20;11:432. doi: 10.1038/s41398-021-01556-8 (PMC8379251; doi:10.1038/s41398-021-01556-8)
Supplement: Supplementary file 1 — Supplementary materials [file 41398_2021_1556_MOESM1_ESM.docx]

**Supplementary materials manuscript Hutschemaekers et al.**

**Details Methods and Materials**

*Heart rate (explorative outcome)*

We adopted HR (as measured with a Polar RS800CX) as a psychophysiological index of the effects of exposure (see supplementary materials for details). The raw data of the R-R (inter-beat) intervals was analyzed using Kubios HRV Analysis Software^1^. Artefacts in R-R data were corrected using the software threshold-based artefact correction (0.25 seconds). Mean HR was calculated for a 2-minute resting period 15 minutes prior to each session and for 2-minute samples during speech delivery, resulting in a maximum of four samples per speech per participant (0-2 min, 2-4 min, 4-6 min, 6-8 min).

*Details on eligibility screening*

The raters conducting the MINI were psychology students who after having fulfilled courses of their bachelor or Master degree did an internship at treatment facility in Nijmegen and who as part of their training underwent a MINI training. The MINI administration was supervised by a registered psychologist, with whom they discussed every MINI administered in biweekly supervision meetings

*Procedure heart rate measurements*

A Polar RS800CX was used to assess resting-state heart rate (HR), i.e., during a 2-minute period before the start of the exposure sessions, and during speech delivery in each session (6-8 minutes). The device uses a transmitter consisting of a polyamide case with electrodes attached to an elastic belt fixated to the chest. Interbeat (R-R) intervals (IBI) were measured at a frequency of 1000 Hz in order to provide a temporal resolution of 1 ms for each R-R interval. The Polar RS800CX has been found to be an accurate, reliable, and valid measure to record heart rate variability ^2–4^. Polar Pro Trainer 5 software was used to transfer the HR recordings to a computer. The raw data was analyzed using Kubios HRV analysis software ^1^. Artefacts in the R-R data were corrected using the threshold-based artefact correction of the Kubios software. This algorithm compares every IBI value to a local average interval. The local average is obtained by median filtering the IBI time series, and thus, the local average is not affected by single outliers in IBI time series. A threshold value of .25 seconds was selected.

Mean HR was calculated for the baseline measurement (2 minutes recorded 15 minutes before each session) and for the 2-minute samples during each exposure, resulting in a maximum of four samples per speech per participant (0-2 minutes, 2-4 minutes, 4-6 minutes, 6-8 minutes). Because not every participant presented a speech lasting more than 6 minutes, we had only 3 HR samples for some participants.

*Statistical analyses HR*

To examine whether enhanced exposure affected psychophysiological reactivity we post-hoc analyzed heart rate responses with the same mixed models as fear levels in session 1 and session 2), including baseline HR as a fixed factor.

**Details results**

*Adverse events (AEs)*

Compared to the placebo (P) group, the testosterone (T) group reported fewer AEs, including mild headache (T: *n* = 1, P: *n* = 4), mild nausea (T: *n* = 0, P: *n* = 2), and mild stomachache (T: *n* = 0, P: *n* = 1). Around 1.5 weeks after the post-treatment assessment, one participant in the T-group, who was on hormonal birth control, reported a breakthrough bleeding lasting several days. On request of her GP, our attending psychiatrist de-blinded her randomization (with the condition remaining blinded for the researchers) three days before the one-month follow-up. No serious AEs were reported.

*Details analyses of the effects of testosterone-administration on exposure (session 1)*

*Fear*

The model showed that the linear and quadratic terms were significant predictors (each added significantly to the model fit). SUD scores reduced over time, confirming that the enhanced exposure resulted in the expected within-session reduction in fear: *Estimate* (linear) = -81.96 (16.76), F(1,50) = 23.89, *p* <.001, *Estimate* (quadratic) = −85.12(13.99), F(1,50) = 36.95, *p* < .001. Of main interest here, was the Group x Time effect. The interaction was not significant: *Estimate*(linear) = -.86(16.76), F(1,51) = .003, *p* = .959, *Estimate*(quadratic) = 7.01(13.99), F(1,51) = .251, *p* = .619, which showed that the fear patterns observed for the enhanced session did not differ per group. Accordingly, both groups followed the same quadratic pattern (Figure S2, panel A).

In the post-hoc model in which the baseline testosterone data were included, the effects of Time were confirmed: *Estimate*(linear) *=* -82.23(16.96), F(1,48) = 23.45, *p* <.001, *Estimate*(quadratic) = −87.07(13.69), F(1,48) = 40.39, p <.001, *Estimate*(cubic) = -23.42(11.54), F(1,47) = 4.09, *p* = .049. No Time x Group interactions were found: *Estimate*(linear) = -1.00(16.96), F(1,48) = .00, *p* = .995, *Estimate*(quadratic) = 8.01(13.69), F(1,47) = .341, *p* = .619, *Estimate*(cubic) = -1.08(11.45), F(1,47) = .01, *p* = .926. We did find a significant Time(quadratic) x Group x Baseline-T effect: *Estimate =* 2.26(.94), *F*(1,48) = 5.72, *p =* .021. The fear patterns recorded per group depended on baseline testosterone levels. For the P group, fear scores showed the same quadratic pattern regardless of basal testosterone: *Estimate = .82*(1.14), *F*(1,24) = .52, *p =* .476, while in the T group fear levels showed a higher peak which then reduced more sharply for the participants with high endogenous testosterone levels, while for the participants with low endogenous testosterone the peak fear levels flattened and reduced less: *Estimate =* -3.73(1.48), *F*(1,24) = 6.32, *p =* .019 (Figure 2, main manuscript, panel A).

*Details analyses of transfer to unenhanced exposure (session 2)*

The model showed that fear levels reduced over time (linear, cubic, and quadratic), confirming that the unenhanced exposure resulted in the expected within-session reduction: *Estimate* (linear) *= -*62.94(15.42), F(1,50) = 16.66, *p* <.001, *Estimate* (quadratic) = −48.32(11.03), F(1,50) = 19.18, p < .001, *Estimate* (cubic) = -36.76(8.90), F(1,50) = 17.01, *p* <.001. Of main interest was the Group x Time effect; a significant interaction was found between Time (quadratic) and Group: *Estimate* = 23.68(11.03), F(1,50) = 4.61, *p =* .037*,* indicating that the fear patterns differed per group. Compared to the P group, the participants in the T group reported higher fear levels throughout the session, with a steeper decline towards the end of the session (see Figure S2, panel B).

*Details HR analyses session 1 and session 2*

*Session 1*

Baseline HR was a significant predictor: *Estimate* *=* .65(.11), F(1,49) = 32.72, *p* <.001, i.e., the higher the baseline HR, the higher the HR during exposure. The linear and quadratic time terms were both significant predictors and each added to the model fit: *Estimate* (linear) = -26.51(6.44), F(1,49) = 16.89, *p* < .001; *Estimate* (quadratic) = 7.50(3.09), F(1,49) = 5.84, *p* = .019. Mean HR during exposure reduced over time, confirming that exposure resulted in the expected within-session reduction. We did not find a significant interaction between Time and Group: *Estimate*(linear) = 5.43(6.44), F(1,49) = .71, *p =* .404*,* *Estimate*(quadratic) = -1.86(3.09), F(1,48) = .36, *p* = .551. The HR patterns in the T group did not differ from those in the P group.

With baseline testosterone in the post-hoc-model we again found Time effects: *Estimate* (linear) *=* -28.07(6.29), F(1,47) = 19.85, *p* < .001, *Estimate*(quadratic) *=* 7.68(3.23), F(1,46) = 5.63, *p* = .022, and the absence of Time x Group interactions were confirmed: *Estimate*(linear) *=* 6.22(6.29), F(1,47) = .97, *p* = .329, *Estimate*(quadratic) *=* -2.44(3.23), F(1,46) = .57, *p* = .456. There was a non-significant trend towards a Time(linear) x Group x Baseline T interaction: *Estimate* =.80(.42), F(1,44) = 3.64*, p =* .063. Partly in line with the fear patterns, the HR reductions following P showed the same slope regardless of baseline T: *Estimate* = .04(.03), F(1,23) = .056, *p* = .461, while after T HRs reduced more so for the participants with higher basal T: *Estimate* = -.12(.06), F(1,21) = 3.89, *p* = .061 (see Figure S3).

*Session 2*

The model showed that baseline HR was a significant predictor: *Estimate* *=* .70(.10), F(1,51) = 43.90, *p* <.001, signifying that the higher the baseline HR, the higher the HR during exposure. The linear time term was a significant predictor. Mean HR during exposure reduced over time, confirming that exposure resulted in the expected within-session reduction: *Estimate =* -12.77(4.59), F(1,50) = 7.70, *p* = .008. We did not find a significant interaction between Time x Group: *Estimate(linear)* = 6.91(4.59), F(1,50) = 2.26, *p =* .139*, Estimate (quadratic)* = -1.82(2.36), F(1,48) = .587, *p =* .447. This means that the HR patterns recorded in session 2 did not differ for the two groups.

The same effects were noted with baseline T in the model: Time(linear): *Estimate* *=* -13.92(4.66), F(1,46) = 8.87, *p* = .005; Time x Group *Estimate*(linear) = 8.11(4.66), F(1,47) = 3.01, *p =* .089, *Estimate*(quadratic) = 1.49(2.38), F(1,45) = .386, *p =* .537. Unlike subjective fear, the psychophysiological HR indices per group were independent of baseline testosterone: Time(linear) x Group x Baseline-T, *Estimate* =.10(.31), F(1,45) = .10, *p* = .759.

**References**

1 Tarvainen, M.P., Niskanen, J.P., Lipponen, J.A., Ranta-aho, P.O. & Karjalainen, P.A. Kubios HRV - Heart rate variability analysis software. *Comput Methods Programs Biomed* 2014; **113**: 210–220.

2 Tsitoglou, K.I., Koutedakis, Y., Dinas, P.C. & Khadka, R. Validation of the Polar RS800CX for assessing heart rate variability during rest , moderate cycling and post-exercise recovery. *F1000 Res* 2019; **7**: 1–13.

3 Vasconcellos, F.V.A. *et al.* Heart rate variability assessment with fingertip photoplethysmography and polar RS800cx as compared with electrocardiography in obese adolescents. *Blood Press Monit* 2015; **20**: 351–360.

4 Williams, D.P. *et al.* Two-week test – retest reliability of the Polar ® RS800CX TM to record heart rate variability. *Clin Physiol Funct Imaging* 2017; **37**: 776–781.

**Figure legends**

**Figure S1***. CONSORT flowchart*

**Figure S2.** SUDs over time as a function of group. The left panel (A) depicts the scores recorded for the enhanced session and the right panel (B) the scores for the second, unenhanced session. In the first session SUDs have a similar pattern over time in both groups. In the second session SUDs show a more reactive pattern with a steeper decline towards the end for the participants in the testosterone group. **Note.** Plots are based on the modeled data, including fixed and random effects. SUD = subjective units of distress.

**Figure S3.** This plot shows the mean heart rates (HRs) during the first exposure session, with placebo [left panel] and with testosterone [right panel]). Mean HRs are displayed over time as a function of baseline testosterone. In order to visualize the interaction effect between baseline Testosterone and Time we divided baseline testosterone in low (-1 SD), medium (mean) and high (+1SD) values. The plot illustrates the finding that high baseline testosterone is associated with a steeper decrease in HR during the testosterone-enhanced exposure. There is no correlation between start HR and baseline testosterone levels for either group (r_placebo_ = -.14, *p* = .507 ; r_testosterone_ = .04 , *p =* .851).
