# Supplementary figures and images for: The enhancing effects of testosterone in exposure treatment for social anxiety disorder: a randomized proof-of-concept trial
Source: Transl Psychiatry. 2021 Aug 20;11:432. doi: 10.1038/s41398-021-01556-8 (PMC8379251; doi:10.1038/s41398-021-01556-8)

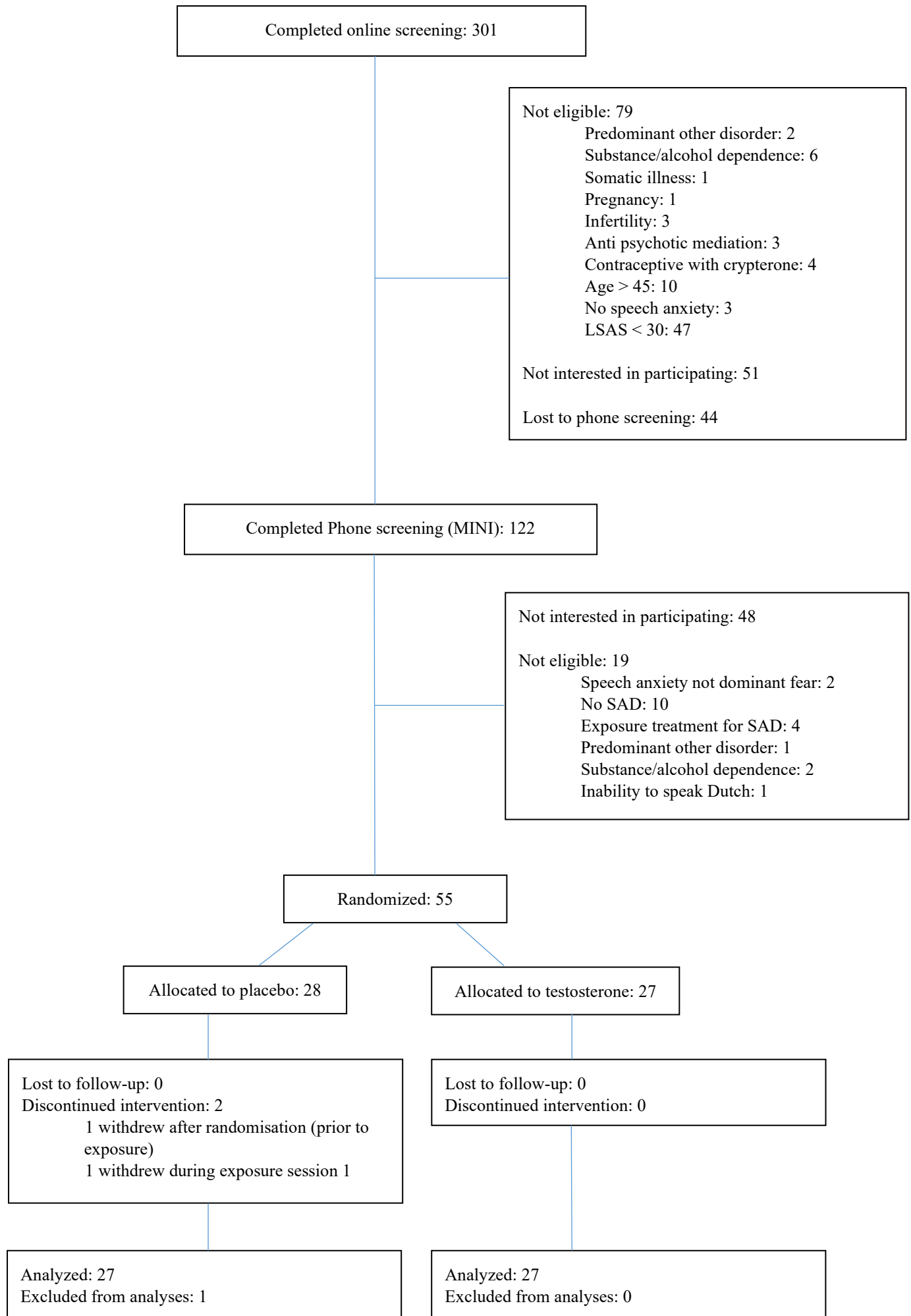

Supplement: Supplementary file 2 — Figure S1 [file 41398_2021_1556_MOESM2_ESM.pdf]

A. Session 1 (enhanced session)

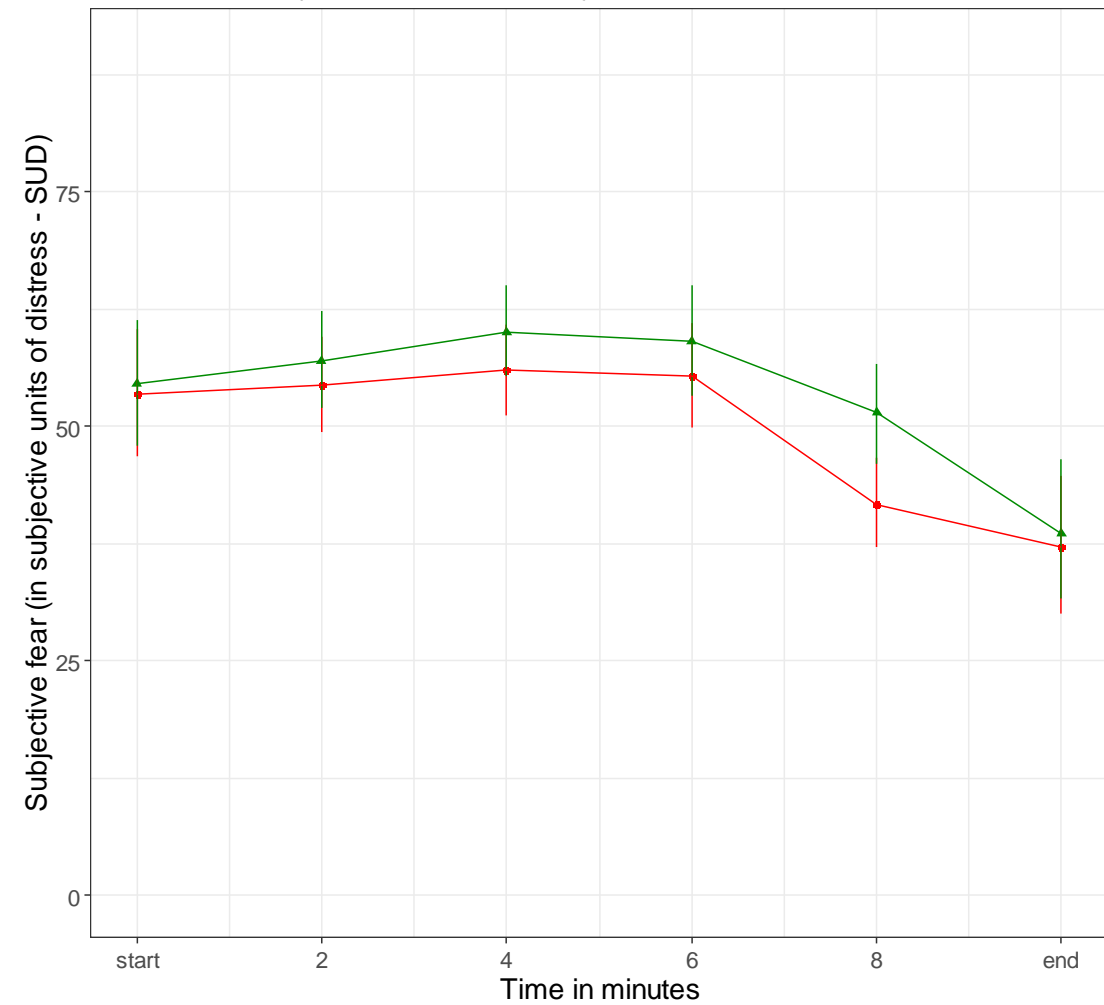

B. Session 2 (unenhaned transfer session)

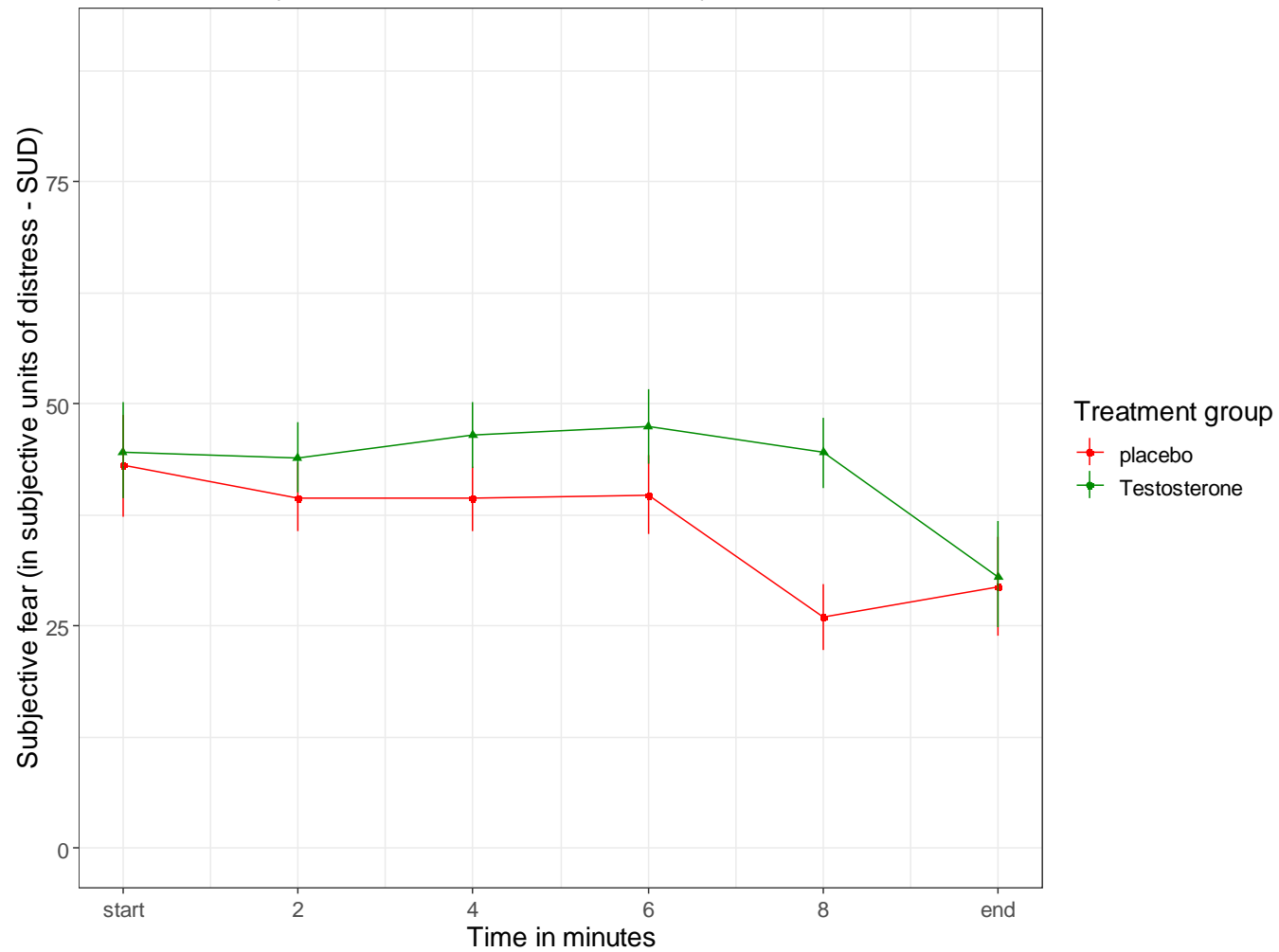

Supplement: Supplementary file 3 — Figure S2 [file 41398_2021_1556_MOESM3_ESM.pdf]

# Session 1 (enhanced session)

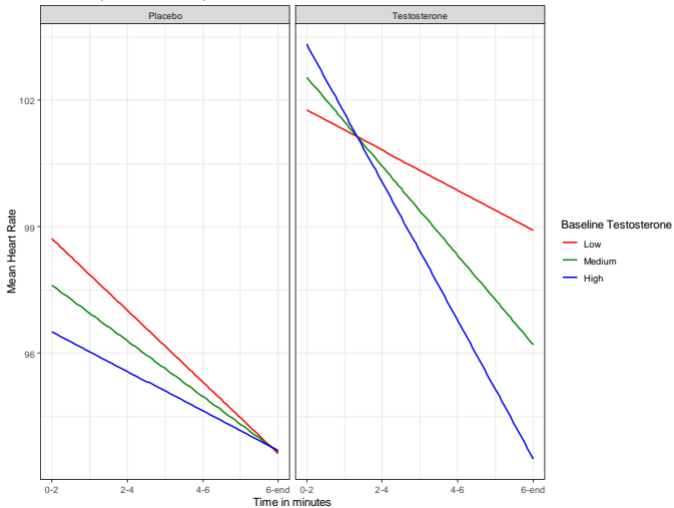

Supplement: Supplementary file 4 — Figure S3 [file 41398_2021_1556_MOESM4_ESM.pdf]
